# Preparation and First Preclinical Evaluation of [^18^F]FE@SNAP: A Potential PET Tracer for the Melanin-Concentrating Hormone Receptor-1 (MCHR1)

**DOI:** 10.3797/scipharm.1306-02

**Published:** 2013-07-01

**Authors:** Cécile Philippe, Lukas Nics, Markus Zeilinger, Eva Schirmer, Helmut Spreitzer, Georgios Karanikas, Rupert Lanzenberger, Helmut Viernstein, Wolfgang Wadsak, Markus Mitterhauser

**Affiliations:** 1 Radiochemistry and Biomarker Development Unit, Department of Nuclear Medicine, Medical University of Vienna, Waehringer Guertel 18–20, 1090 Vienna, Austria.; 2 Department of Pharmaceutical Technology and Biopharmaceutics, University of Vienna, Althanstraße 14, 1090 Vienna, Austria.; 3 Department of Nutritional Sciences, University of Vienna, Althanstraße 14, 1090 Vienna, Austria.; 4 Department of Drug and Natural Product Synthesis, University of Vienna, Althanstraße 14, 1090 Vienna, Austria.; 5 Department of Psychiatry and Psychotherapy, Medical University of Vienna, Waehringer Guertel 18–20, 1090 Vienna, Austria.; 6 Department of Inorganic Chemistry, University of Vienna, Waehringer Straße 42, 1090 Vienna, Austria.

**Keywords:** MCHR1, Fluorine-18, PET, SNAP-7941, Radioligand

## Abstract

The melanin-concentrating hormone (MCH) system is a new target for the treatment of human disorders. Since the knowledge of the MCH system’s involvement in a variety of pathologies (obesity, diabetes, and deregulation of metabolic feedback mechanism) is based on *in vitro* or preclinical studies, a suitable positron emission tomography (PET) tracer needs to be developed. We herein present the preparation and first preclinical evaluation of [^18^F]FE@SNAP – a new PET tracer for MCH receptor-1 (MCHR1). The synthesis was performed using a microfluidic device. Preclinical evaluation included binding affinity, plasma stability, plasma free fraction, stability against the cytochrome P-450 (CYP450) system using liver microsomes, stability against carboxyl-esterase, and methods to assess the penetration of the blood-brain barrier (BBB) such as logD analysis and immobilized artificial membrane (IAM) chromatography. Levels at 374 ± 202 MBq [^18^F]FE@SNAP were obtained after purification. The obtained *K*_d_ value of [^18^F]FE@SNAP was 2.9 nM. [^18^F]FE@SNAP evinced high stability against carboxylesterase, CYP450 enzymes, and in human plasma. LogD (3.83) and IAM chromatography results (P_m_=0.51) were in the same range as for known BBB-penetrating compounds. The synthesis of [^18^F]FE@SNAP was reliable and successful. Due to high binding affinity and stability, [^18^F]FE@SNAP is a promising tracer for MCHR1.

## Introduction

**Fig. 1:**
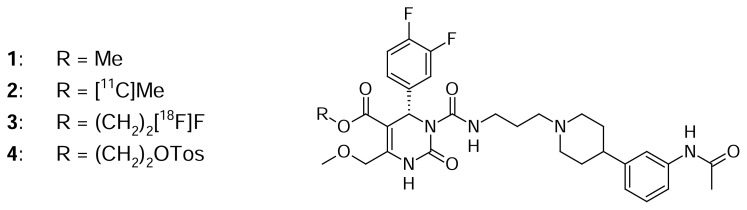
SNAP-7941 derivatives **1–4** (**1**: SNAP-7941; **2**: [^11^C]SNAP-7941; **3**: [^18^F]FE@SNAP; **4**: Tos@SNAP)

Melanin-concentrating hormone (MCH) is a cyclic nonadeca-peptide, predominantly expressed in the lateral hypothalamus and zona incerta [1, 2]. Besides, it is also found in peripheral organs and tissues, such as the pancreas [3], colonic epithelial cells [4], or adipocytes [5, 6]. The biological function of MCH is mediated by two G-protein-coupled receptors, MCH receptor-1 and -2 (MCHR1 [7–10] and MCHR2 [11–14]). MCH plays a key role in energy homeostasis, e.g. the control of food intake and body weight [15, 16]. Furthermore, it is involved in diabetes, gut inflammation, and adiposity [3–6]. The widespread distribution of MCH and its receptors and the involvement in a variety of pathologies makes the MCH system an interesting new target for the treatment of human disorders. Several MCHR1 antagonists were presented in the last decade; some of them have been entered in clinical trials for the treatment of obesity [17] and some are being discussed as potential anti-diabetic drugs [18]. However, to ensure confidence in preclinical to clinical translation of MCHR1 pharmacology and for further studies on the involvement and distribution of MCHR1 in energy homeostasis, a suitable positron emission tomography (PET) tracer needs to be developed. PET is a non-invasive technique for visualizing molecular effects directly *in vivo*. Borowsky et al. [19] presented the evaluation of the very potent MCHR1 antagonist SNAP-7941 ((+)-methyl (4S)-3- {[(3-{4-[3-(acetylamino)phenyl]-1-piperidinyl}propyl)amino]carbonyl}-4-(3,4-difluorophenyl)-6-(methoxymethyl)-2-oxo-1,2,3,4-tetrahydro-5-pyrimidinecarboxylate hydrochloride, **1**, Fig. 1) (*K*_d_=0.18 nM, evaluated on Cos-7 cells expressing the human MCHR1 (hMCHR1) [19]). We previously reported the successful radiosyntheses of its radiolabeled analogues, [^11^C]SNAP-7941 [20] (**2**, Fig. 1) and [^18^F]FE@SNAP [21] (**3**, Fig. 1). The present work focuses on:

1. the up-scaling, purification, and formulation of [^18^F]FE@SNAP, and
2. on the *in vitro* assessment of its potential as a PET tracer through preclinical evaluation of its main biological and physicochemical properties.

## Results

### Radiochemistry

From a single synthesis in the microfluidic system 374 ± 202 MBq (range: 98–662 MBq) [^18^F]FE@SNAP were obtained after purification (n=6). Radiochemical purity always exceeded 98%. Subsequently, 3.1 ± 0.5 μg FE@SNAP were detected in the final product solution. Precursor mass was below the limit of detection (< 0.5 μg/mL). Specific radioactivity was 24.8 ± 12 GBq/μmol at the end of synthesis (EOS). Residual solvent analysis revealed < 10 ppm acetonitrile and no other impurities. Osmolality was 222 ± 4 mosmol/kg and pH was 7.4 ± 0.2.

### Biological Evaluation

**Fig. 2:**
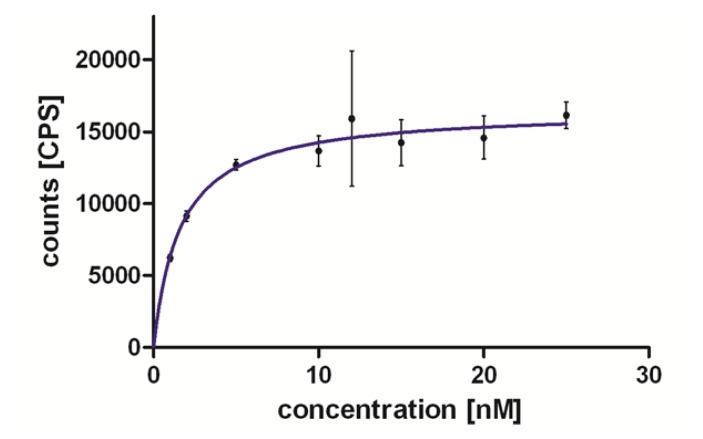
Specific binding of [^18^F]FE@SNAP on the hMCHR1. If not visible, error bars are within the margin of the symbols.

The binding experiments on hMCHR1 revealed a *K*_d_ of 2.9 nM (n=2) for [^18^F]FE@SNAP (Fig. 2). Preliminary competition binding experiments against [^125^I]MCH on hMCHR2 showed poor binding of FE@SNAP (*K*_i_ > 1000 nM).

The degradation of [^18^F]FE@SNAP in human plasma (n=6) was 0.48 ± 0.5% after 0 min and 3.87 ± 3.9% after 120 min. In rat plasma (n=11) the degradation was 32.44 ± 33.5% initially, and after 120 min, [^18^F]FE@SNAP was completely metabolized. The formation of a radioactive hydrophilic metabolite could be observed.

The plasma free fraction (f_1_) of [^18^F]FE@SNAP was 12.6 ± 0.2% in human plasma (n=3). Due to the fast metabolism of [^18^F]FE@SNAP in rat plasma, it was not possible to determine f_1_ in that medium.

**Fig. 3:**
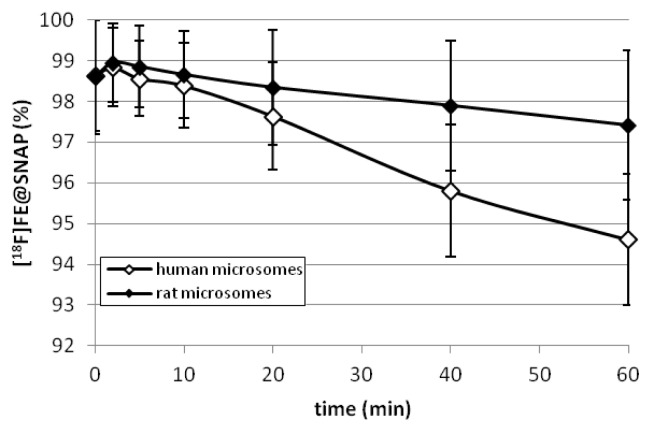
Degradation of [^18^F]FE@SNAP by rat and human liver microsomes.

The enzymatic degradation of [^18^F]FE@SNAP by CYP450 after 60 min was 5.39 ± 1.6% using human liver microsomes (n=4) and 2.59 ± 1.8% using rat liver microsomes (n=4) (Fig. 3).

**Fig. 4:**
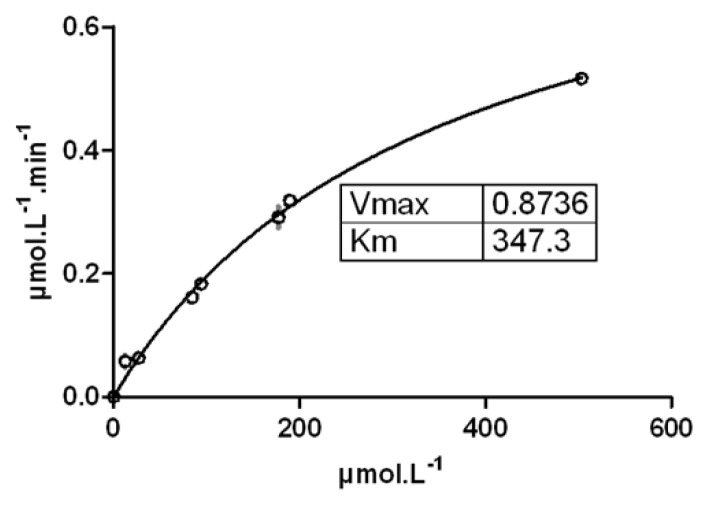
Michaelis-Menten saturation curve of FE@SNAP against carboxylesterase. If not visible, error bars are within the margin of the symbols.

The Michaelis-Menten constant (K_m_) of FE@SNAP was 347.3 μM and the limiting velocity (V_max_) was 0.874 μM/min (Fig. 4).

### Physicochemical Parameters

The logD value of FE@SNAP was 3.83 ± 0.1 (n=3) and the value of the permeability through the membrane (P_m_) was 0.51 ± 0.1 (n=3). Preliminary experiments on hMCHR2 showed poor binding of FE@SNAP (*K*_i_ > 1000 nM).

## Discussion

Due to the low density of the MCH receptors in the human brain (B_max_=5.8 ± 0.3 fmol/mg, [22]), a high binding affinity in a low nanomolar range of [^18^F]FE@SNAP is mandatory. [^18^F]FE@SNAP evinced a high affinity in saturation binding assays (*K*_d_=2.9 nM). Moreover, FE@SNAP revealed high selectivity for hMCHR1 (*K*_i_ on hMCHR2 > 1000 nM) in competitive binding assays.

Starting from 29 ± 4 GBq [^18^F]fluoride, 374 ± 202 MBq (2.6 ± 1.5% at the end of bombardment (EOB)) [^18^F]FE@SNAP were obtained. Three different circumstances led to this unexpected low radiochemical yield:

1. Due to incomplete priming of the solution into the loop to guarantee bubble-free filling (which is a systematic problem in the microfluidic system that was used), 16.1 ± 0.3% of the activity remained in the concentrator vial of the microfluidic system after azeotropic drying and was not accessible for the synthesis [23].
2. Furthermore, 8.0 ± 3.5% remained in the lines and was thereby not accessible for the reaction [23].
3. The syntheses were performed in the discovery mode of the microfluidic system. There, approximately half of the amount of activity from the loop was used for the synthesis. The residual activity was kept as a reserve in the loop to have the option for a second consecutive synthesis.

However, 374 ± 202 MBq [^18^F]FE@SNAP were sufficient for any subsequent preclinical evaluation studies. Higher radiochemical yields can be achieved using the sequence mode of the microfluidic system.

The tested quality control parameters of the physiologically formulated [^18^F]FE@SNAP solution were in accordance with the standards for human application. Specific activity was relatively low (24.8 ± 12 GBq/μmol). Higher specific activities are expected for future syntheses with higher yields. We note that no conversion of [^18^F]FE@SNAP could be achieved using conventional synthesizing modules [21].

We evaluated the stability of [^18^F]FE@SNAP not only in human, but also in rat tissues (plasma, liver microsomes) to be prepared for species differences in future small animal PET experiments. [^18^F]FE@SNAP was highly stable against human and rat liver microsomes (consisting of the multienzyme complex cytochrome P-450: 5.39 ± 1.6% (human) and 2.59 ± 1.8% (rat) decomposition after 60 min) and in human plasma (only 3.87 ± 3.9% metabolism after 120 min). In contrast, [^18^F]FE@SNAP was completely metabolized in rat plasma within 120 min.

The amount of unbound (free) [^18^F]FE@SNAP (f_1_=12.6 ± 0.2%) in human plasma should be sufficient for potential future clinical PET-studies targeting the brain. In comparison: the 5HT_1A_ ligand [carbonyl-^11^C]WAY-100635 has a plasma free fraction of 5.8 ± 0.2% [24].

Porcine carboxylesterase was used to assess MMK, due to its wide use as a biochemical model for *in vitro* studies [25]. With a K_m_ of 347.3 μM, FE@SNAP again showed very high stability.

For the prediction of blood-brain barrier (BBB) penetration, the lipophilicity expressed as logD was measured in the first step. Since the logP/logD values were shown to be poor predictors for BBB penetration [26], immobilized artificial membrane (IAM) chromatography was additionally performed. Under the modified conditions from Tavares et al. [27], FE@SNAP (P_m_=0.51) is situated well in between β-CIT (P_m_=0.31) and DASB (P_m_=1.23) – two known BBB penetrating compounds. Therefore, considering only passive diffusion, a penetration through the BBB seems possible.

Compared to [^11^C]SNAP-7941, which we evaluated previously [34], [^18^F]FE@SNAP was only accessible via microfluidic chemistry. Both tracers evinced a high binding affinity and selectivity to hMCHR1 and a high metabolic stability in human plasma and against liver microsomes and carboxylesterase. The rapid enzymatic degradation in rat plasma was also observed with [^11^C]SNAP-7941. These similarities confirm the analogy of methyl- and fluoroethylesters described by Nics et al. [32]. The logD and P_m_ values of both tracers were similar too. Summing it all up, [^18^F]FE@SNAP is comparable to [^11^C]SNAP-7941 in its biological behaviour with the advantage of the longer-lived radioisotope ^18^F (*t*_1/2_=110 min) instead of ^11^C (*t*_1/2_=20 min).

## Conclusion

The synthesis of [^18^F]FE@SNAP yielded sufficient amounts (374 ± 202 MBq) for use in subsequent preclinical evaluations. Our main criteria to pursuit the evaluation of [^18^F]FE@SNAP as a PET tracer for MCHR1 were:

➢ a high binding affinity to MCHR1 in a low nanomolar range,
➢ high metabolic stability to assure enough intact tracer for the visualization of MCHR1-specific tissues and
➢ reasonable lipophilicity to expect BBB penetration.

FE@SNAP binds to hMCHR1 in a nanomolar range (K_d_=2.9 nM) and is highly selective to this receptor subtype. It showed very high stability against porcine carboxylesterase, the cytochrome P-450 fraction of human and rat liver microsomes and in human plasma. Furthermore, the human plasma free fraction (f_1_=12.6 ± 0.2%) is high enough for potential brain imaging. The fact that decomposition in rat plasma is complete within 120 min has to be considered for further preclinical studies in rats. As IAM chromatography experiments showed comparable behavior to known BBB-penetrating compounds, passive BBB penetration is possible. Collectively, [^18^F]FE@SNAP is a promising tracer for MCHR1, and further preclinical evaluation steps (e.g. autoradiography and small-animal PET) will thus elucidate its potential.

## Experimental

### General

#### Materials

[^18^F]fluoride was produced via the ^18^O(p,n)^18^F reaction in a GE PET trace cyclotron (16.5-MeV protons; GE Medical Systems, Uppsala, Sweden). H_2_^18^O (HYOX18; > 98%) was purchased from Rotem Europe (Leipzig, Germany). Typical beam currents were 48–52 μA and irradiation was stopped as soon as the desired activity level was reached (approx. 25–30 GBq). Anion-exchange cartridges (PS-HCO_3_) for [^18^F]fluoride trapping were obtained from Macherey-Nagel (Dueren, Germany). The precursor compound (Tos@SNAP; **4**, Fig. 1) and the reference standard (FE@SNAP) were synthesized in cooperation with the Department of Drug and Natural Product Synthesis of the University of Vienna (Austria) [28, 29]. Solid phase extraction (SPE) cartridges SepPak® C18-plus and the Oasis HLB 6cc Vac (200 mg) were purchased from Waters (Waters® Associates, Milford, MA, USA). Sterile water Ecotainer® and 0.9% saline solution were purchased from B. Braun (Melsungen, Germany). 3% saline solution was obtained from a local pharmacy (Landesapotheke Salzburg, Austria). A 125 mM phosphate buffer was prepared by dissolving 0.224g sodium dihydrogen phosphate-monohydrate and 1.935g disodium hydrogen phosphate-dihydrate (both from Merck, Darmstadt, Germany) in 100 mL of sterile water. Phosphate-buffered saline (PBS) concentrate (10:1) was obtained from Morphisto (Frankfurt, Germany). NADPH-regenerating system solution-A and solution-B were obtained from BD Biosciences (Bedford, MA, USA). Acetonitrile, acetic acid, tetrahydrofuran (THF) anhydrous, methanol, ethylendiaminetetraacetic acid (EDTA), bacitracin, bovine serum albumin, and porcine liver carboxylesterase (EC 3.1.1.1) were purchased from Sigma Aldrich (Vienna, Austria). Ammonium acetate, acetonitrile (for DNA synthesis, ≤ 10 ppm H_2_O), Krypotofix 2.2.2, K_2_CO_3_, MgCl_2_, Tris(hydroxymethyl)amino-methane (Tris), triphenylene, toluol, and ethanol were purchased from Merck (Darmstadt, Germany). Pooled human liver microsomes (Lot No. 34689), pooled male rat liver mircosomes (Lot No. 85157), and pooled female rat liver microsomes (Lot No. 59232) (both from Sprague Dawley rats) were purchased from BD Biosciences (Woburn, MA, USA). The male and female rat liver microsomes were homogenized. Pooled lithium-heparinised human plasma (No. IPLA-N) and pooled lithium-heparinised rat plasma (No. IRT-N) were purchased from Innovative Research (Novi, MI, USA). Centrifugal Filter Units (Centrifree®-30K) were purchased from Merck Millipore (Tullagreen, Ireland). [^125^I]MCH and CHO-K1 cell membranes expressing hMCHR1/hMCHR2 were purchased from PerkinElmer (Waltham, MA, USA). The vials for the binding affinity assay were purchased from Beckman Coulter Inc. (Brea, CA, USA; Bio-Vial™, 4 mL, 14 × 55 mm) and from Seton Scientific (Petaluma, CA, USA; Open-Top Centrifuge Tubes Polyclear, 13 × 64 mm). A semi-preparative high-performance liquid chromatography (HPLC) column (Chromolith® SemiPrep RP-18e; 100-4.6 mm), analytical HPLC column (LiChroCART® 250-4 mm), and the column for metabolic stability testing (Chromolith® Performance RP-18e; 100-4.6 mm precolumn: Chromolith® Guard Cartridge RP-18e; 5-4.6 mm) were purchased from Merck (Darmstadt, Germany). A gas chromatography capillary column (forte GC Capillary Column ID-BP20; 12 m × 0.22 mm × 0.25 μm) was purchased from SGE Analytical Science Pty. Ltd. (Victoria, Australia). IAM (immobilized artificial membrane) chromatography was performed using an IAM.PC.DD2 column (15 cm × 4.6 mm) (Regis Technologies Inc., Morton Grove, IL, USA). The ODP-50 column for logD measurement was purchased from Shodex™ (Showa Denko Europe GmbH, Munich, Germany).

#### Instrumentation

The radiosynthesis of [^18^F]FE@SNAP was carried out within an Advion NanoTek® unit (Ithaca, NY, USA) comprising a concentrator unit (CE) and a liquid flow reaction unit (LF) with dedicated control software (Advion, version 1.4). Microreactors were made of fused silica tubing (ID, 0.1 μm; length 2.0 m), wound up and held in a brass ring, and filled with a thermoresistant polymer to hold the tubing in place. The purification of the resulting crude product solution and the final formulation of [^18^F]FE@SNAP was carried out within a Nuclear Interface® PET synthesizer (GE Medical Systems, Uppsala, Sweden) remote controlled via GINAstar software (Raytest Isotopenmessgeräte GmbH, Straubenhardt, Germany) installed on a standard PC. Analytical HPLC was performed using an Agilent system (Boeblingen, Germany) consisting of an autosampler 1100, a quartenary pump 1200, a diode array detector 1200 (operated at 254 nm), and a lead-shielded BGO-radiodetector. The osmolality was measured using a Wescor osmometer Vapro® 5600 (Sanova Medical Systems, Vienna, Austria), pH was measured using a WTW inoLab 740 pH meter (WTW, Weilheim, Germany). Gas chromatography was performed using a 430-GC system (Burker Daltonik GmbH, Bremen, Germany). For the binding experiments, a Sorvall Ultracentrifuge Combi OTD (Thermo Fisher Scientific Inc, Waltham, MA, USA) and a 2480 WIZARD^2^ Automatic Gamma Counter (PerkinElmer, Waltham, MA, USA) were used. For the stability experiments, sample incubation was conducted within a Thermomixer compact from Eppendorf® (Vienna, Austria) and sample centrifugation with a Universal 30 RF centrifuge (Hettich, Tuttlingen, Germany). The same centrifuge was used for the determination of the plasma free fraction.

### Radiochemistry

#### Radiosynthesis

**Fig. 5:**
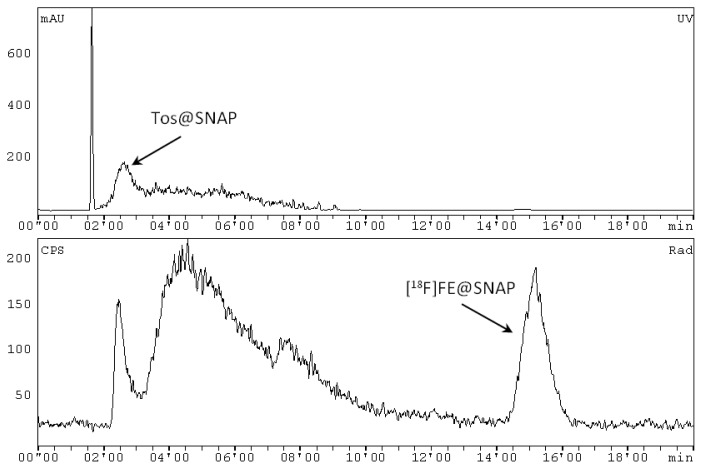
Representative semi-preparative HPLC chromatogram of the reaction solution of [^18^F]FE@SNAP. Top chromatogram: UV channel (mAu, milli Absorbance Unit). Bottom chromatogram: radioactivity channel (NaI); (CPS, counts per second).

The azeotropic drying of the cyclotron-produced [^18^F]fluoride and the radiosynthesis of [^18^F]FE@SNAP were carried out within a microfluidic system (Advion NanoTek®) as described in detail elsewhere [21]. Briefly, n.c.a [^18^F]fluoride (25–30 GBq) was trapped on an anion exchange cartridge (PS-HCO3) and released with a solution containing Kryptofix 2.2.2 (4,7,13,16,21,24-hexaoxa-1,10-diazabicyclo[8.8.8]hexacosane; 10 mg, 26.6 μmol) and potassium carbonate (2.25 mg, 16.6 μmol) in acetonitrile/water (70/30 v/v; V=0.5 mL). Iterative azeotropic drying was performed at 110°C by the addition of three times 300 μL dry acetonitrile. Subsequently, the dried [^18^F]fluoride-aminopolyether was dissolved in 500 μL acetonitrile. 150–200 μL of Tos@SNAP (6 mg/mL in acetonitrile (for DNA synthesis)) and the same volume of the [^18^F]fluoride-aminopolyether in acetonitrile (final precursor concentration: 3mg/mL) were simultaneously pushed through the microreactor at 170°C with a total flow rate of 170 μL/min. Subsequently, the crude product solution was swept out of the microreactor with a defined volume of 200 μL acetonitrile. The crude product solution was transferred into the Nuclear Interface® synthesizer unit, quenched with 1 mL water and subsequently injected into the semi-preparative HPLC column (mobile phase: (water/acetic acid 97.5/2.5 v/v; 2.5 g/L ammonium acetate; pH 3.5)/acetonitrile 75/25 v/v; flow: 8 mL/min, after 9 min: 10 mL/min). The chromatograms were registered using a UV-detector (245 nm) and a NaI radioactivity detector in series. The retention times were 2′20–3′10 (*k*′=0′16–0′63) for Tos@SNAP and 14′05–16′35 min (*k*′=5′11–6′11) for [^18^F]FE@SNAP (Fig. 5). The [^18^F]FE@SNAP fraction was cut and diluted with 100 mL water. This aqueous product solution was then pushed through a C18 SPE cartridge. After washing with 10 mL water, the pure product was eluted with 1.5 mL ethanol and 5 mL 0.9% saline solution. The formulation was done with an additional 9 mL of physiological saline (0.9%), 1 mL of saline solution (3%), and 1 mL phosphate buffer (125 nM). Hence, the final total volume was 17.5 mL. For the stability and binding affinity experiments, [^18^F]FE@SNAP was eluted from the SPE cartridge with only 1 mL ethanol and 0.5 mL water in order to enhance the activity concentration in the product solution.

#### Quality Control

Chemical and radiochemical impurities were detected using an analytical HPLC (mobile phase: 0.1 M ammonium acetate/acetonitrile 60/40 v/v; flow: 1 mL/min). The retention time of [^18^F]FE@SNAP was 11.8–12.5 min (*k*′=4.9–5.3). The chemical identity of [^18^F]FE@SNAP was determined by co-injection of the unlabeled reference compound, FE@SNAP. The physiologically formulated product solutions were further checked on residual solvents (analyzed by GC), osmolality, and pH (checked with dedicated equipment).

### Biological Evaluation

#### Binding Affinity

The method used was conducted according to Mashiko et al. [30] with minor modifications. CHO-K1 cell membranes expressing hMCHR1 (10 μg/mL) were dissolved in 500 μL and a 50 mM Tris buffer (pH 7.4) (containing 10 mM MgCl_2_, 2 mM EDTA, 0.1% bacitracin and 0.2% BSA). For the evaluation of the equilibrium dissociation constant (*K*_d_) of [^18^F]FE@SNAP, several concentrations (0–500 nM) of [^18^F]FE@SNAP were added. The membranes were incubated in vials at room temperature for 120 min. Bound and free fractions of the radioligand were separated by centrifugation at 40.000 × *g* for 20 min. The supernatants were removed into new vials. The pellets were washed with 800 μL of the ice cold Tris buffer, which was added to the supernatant, and the pellets were dissolved in 1300 μL of the Tris buffer. The radioactivity in the vials was measured by a Gamma Counter. The *K*_d_ values were calculated by using GraphPad Prism software Version 5.0 (La Jolla, CA, USA).

#### Plasma Stability

The stability of [^18^F]FE@SNAP in human and rat plasma was determined according to Nics et al. [31]. 1800 μL of lithium-heparinized plasma (rat and human, respectively) were pre-incubated under physiological conditions (PBS, pH 7.4, 37°C) in a shaking incubator for 5 minutes. 36 μL [^18^F]FE@SNAP (corresponding to 2% ethanol v/v in the total volume) were added and the plasma vial was vortexed for at least 10 seconds. After defined points in time (0 and 120 min), 500 μL of the incubation-mixture were added to a preconditioned (with 5 mL methanol followed by 5 mL water) SPE-cartridge (Oasis). The cartridge was then eluted into a collection tube, washed with 5 mL of 5% methanol in water (v/v) into a second tube, and eluted with 3 mL of THF into a third tube. 20 μL of the eluate-solution of tube two and three were injected into the analytical HPLC (mobile phase: (water/acetic acid 97.5/2.5 v/v; 2.5 g/L ammonium acetate; pH 3.5)/acetonitrile 70/30 v/v; flow: 2mL/min).

#### Plasma Free Fraction

The method used was modified from Parsey et al. [24]. 1 mL of heparinized plasma (rat and human, respectively) were mixed with 10–50 μL [^18^F]FE@SNAP. 200 μL aliquots were pipetted into the centrifugal filter units and the total radioactivity was measured in a Gamma Counter. After the centrifugation step (2.000 × *g*, 50 min), 50 μL of the obtained filtrate was back-measured for radioactivity. For the determination of the plasma free fraction (f_1_), the ratio of filtrate to total activity concentration was calculated.

#### Stability Against Liver Microsomes (CYP450)

The method used was described by Nics et al. [31]. Briefly, liver microsomes (pooled from human or rat origin) were pre-incubated under physiological conditions (PBS, pH 7.4, 37°C) with a NADPH-generating system (solution-A: NADP+, glucose-6-phosphate and magnesium-chloride in H_2_O and solution-B: glucose-6-phosphate dehydrogenase in sodium citrate) for 5 min. 6 μL of [^18^F]FE@SNAP, which correspond to 2% ethanol (v/v) in the total volume, were added. Enzymatic reactions were stopped after defined points in time (0, 2, 5, 10, 20, 40, and 60 min) by adding the same amount of ice-cold acetonitrile/methanol (10:1). The mixtures were vortexed, followed by a centrifugation step (23.000 × *g*, 5 min). Aliquots of the obtained supernatant were analyzed by an analytical HPLC (for conditions see Plasma Stability).

#### Stability Against Carboxylesterase

The method used was slightly modified from Nics et al. [32]. Incubations of different amounts (10, 30, 50, 70, 100, 200 μg/ml) of FE@SNAP were accomplished with a constant quantity of 80 International Units (I.U.) of porcine carboxylesterase under physiological conditions (PBS, pH 7.4, 37°C). The use of selected concentrations of FE@SNAP was based on an optimal choice to create Michaelis-Menten kinetics (MMK). 35 μl of the incubation-mixture were stopped after defined points in time (0, 60, 120, 180, and 240 min) by adding the same amount of ice-cold acetonitrile/methanol (10:1) and then vortexed. After centrifugation of the reaction mixtures (23.000 × *g*, 5 min), 20 μL of the obtained supernatant were analyzed by analytical HPLC (for conditions see Plasma Stability). The MMK of FE@SNAP was calculated by using GraphPad Prism software Version 5.0 (La Jolla, CA, USA).

### Physicochemical Parameters

#### LogD Analysis

LogD values were determined using an HPLC-based assay according to Donovan and Pescatore [33]. A cocktail of two internal standards (toluene and triphenylene) with known logD and *k*′ values and FE@SNAP in methanol were injected into a short polymeric ODP-50 column. A linear gradient from 10% methanol/90% phosphate buffer (pH 7.4) to 100% methanol within 9.4 min at a flow rate of 1.5 mL/min was applied. Detection was performed at 260 nm and 285 nm.

#### IAM Chromatography

IAM chromatography was modified from Tavares et al. [29]. A 0.01 M phosphate buffer (pH 7.0) and acetonitrile (ranging from 50% to 35%, v/v) were used as the mobile phase at a flow rate of 1 mL/min. FE@SNAP was injected onto the IAM column. As a result, the permeability through the membrane (P_m_) was calculated and compared with the P_m_ of the known BBB-penetrating compounds (DASB, β-CIT) as external standards.
